# *Tricholoma matsutake* Aqueous Extract Induces Hepatocellular Carcinoma Cell Apoptosis via Caspase-Dependent Mitochondrial Pathway

**DOI:** 10.1155/2016/9014364

**Published:** 2016-11-28

**Authors:** Yanzhen Wang, Yiling Chen, Xinrui Zhang, Guangsheng Cai, Shengshu An, Xue Wang, Lirong Teng, Di Wang

**Affiliations:** ^1^Zhuhai College of Jilin University, Jilin University, Zhuhai 519000, China; ^2^School of Life Sciences, Jilin University, Jilin 130012, China; ^3^Southern Research Institute, Jilin University, Zhuhai 519000, China

## Abstract

*Tricholoma matsutake*, one of widely accepted functional mushrooms, possesses various pharmacological activities, and its antitumor effect has become an important research point. Our study aims to evaluate the cytotoxicity activities of* T. matsutake* aqueous extract (TM) in HepG2 and SMMC-7721 cells. In* in vitro* experiments, TM strikingly reduced cell viability, promoted cell apoptosis, inhibited cell migration ability, induced excessive generation of ROS, and caused caspases cascade and mitochondrial membrane potential dissipation in hepatocellular carcinoma cells. In* in vivo* experiments, 14-day TM treatment strongly suppressed tumor growth in HepG2 and SMMC-7721-xenografted nude mice without influence on their body weights and liver function. Furthermore, TM increased the levels of cleaved poly-ADP-ribose polymerase (PARP), Bad, and Bax and reduced the expressions of B-cell lymphoma 2 (Bcl-2) in treated cells and tumor tissues. All aforementioned results suggest that caspase-dependent mitochondrial apoptotic pathways are involved in TM-mediated antihepatocellular carcinoma.

## 1. Introduction

As the most common organ malignancy worldwide, hepatocellular carcinoma (HCC) generally carries a poor prognosis [1]. According to the latest statistics, about 600,000 patients are diagnosed with HCC around the world each year, and some of them die within 7-8 months after diagnosis [2]. Till now, under advanced medical situation, multiple therapeutic options have been applied for HCC therapy; however, these options show various adverse effects including immune system disorders and liver toxicity [3]. Therefore, more effective alternative therapies or medicines with fewer adverse effects for HCC curing are urgently required.

Natural products, with low toxicity and strong pharmacological activities, have become one of the most popular strategies for antitumor agent development [4]. In our group, we have successfully confirmed the proapoptotic properties of* Cordyceps militaris* in hepatocellular carcinoma and breast cancer cells related to caspase-dependent mitochondrial pathway [5].* Tricholoma matsutake*, a highly valued ectomycorrhizal mushroom, exhibits various biological activities such as antimicroorganism, immunostimulation, and antioxidation properties [6]. It has been confirmed that* T. matsutake* strongly inhibits HeLa and HepG2 cell proliferation [7]. In human promyelocytic leukemia cells,* T. matsutake* induces significant damage by activating caspase-related pathway [8]. In* in vivo* mouse models,* T. matsutake* polysaccharides suppress S180 tumor growth, which is believed to be a consequence of the stimulation on cell-mediated immune responses [9]. Although the antitumor effects of* T. matsutake* have already been clarified in several previous researches, its underlying mechanisms of antihepatocellular carcinoma are still unknown.

During cell apoptosis process, abnormal alternations on oxidation system, mitochondrial function, and proapoptotic and antiapoptotic protein levels were observed [10–12]. Mitochondria apoptosis, a well-known death signaling pathway, accompanies mitochondrial depolarization, cytochrome C (Cyt C) overrelease, and caspase-3 activation. Initiator caspase, especially caspase-8 and caspase-9, can catalyze proteolytic maturation of caspase-3, which is recognized as an important effector protease [13]. Interestingly, in extrinsic apoptosis, the activation of caspase-8 increases mitochondrial membrane permeability [14]. On the other hand, hyperlevel of oxidative stress leads to the modification of amino acid residues thereby causing DNA mutations and cell apoptosis [15]. The overgeneration of reactive oxygen species (ROS) causes intracellular oxidative stress and further aggravates mitochondrial depolarization [16].

Based on these encouraging results, the purpose of this study aims to investigate the antihepatocellular carcinoma activity of* T. matsutake* in HepG2 and SMMC-7721 cells systematically. Through* in vitro* and* in vivo* experiments, the proapoptotic effects of* T. matsutake* and underlying mechanisms related to mitochondrial apoptotic pathways were explored.

## 2. Materials and Methods

### 2.1. *T. matsutake* Aqueous Extract Preparation

*T. matsutake* mycelium obtained via liquid submerge fermentation [17] was extracted at 95°C for 3 h in double distilled (DD) water. After concentration using evaporator, the extract was freeze-dried and named TM for further experiments. The content of polysaccharides and total proteins was detected via phenol-sulfuric acid method [18] and Kjeldahl method [19]. TM contains 29.9% of polysaccharides and 19.6% of total proteins.

### 2.2. Cell Culture

Human HCC cell lines HepG2 (CRL-11997; ATCC, USA) and SMMC-7721 (BNCC33; CCTCC, China) were maintained in Dulbecco's Modified Eagle Media (DMEM) supplemented with 10% fetal bovine serum (FBS), 100 units/mL penicillin, and 100 *μ*g/mL streptomycin under a humidified atmosphere containing 5%/95% CO2/air at 37°C. All reagents were obtained from Invitrogen, USA.

### 2.3. MTT Assay

To test the effect of TM on cell viability, a quantitative colorimetric assay with 3-(4,5)-dimethylthiahiazo(-z-y1)-3,5-di-phenytetrazoliumbromide (MTT) was applied. Briefly, HepG2 and SMMC-7721 cells were seeded into 96-well plates at 1 × 10^4^ cells in 100 *μ*L of medium per well. After incubation with 1–5 mg/mL of TM for 24 h and 48 h, cells were incubated with 0.5 mg/mL of MTT for 4 h at 37°C in darkness. After removing the culture medium, 100 *μ*L of dimethylsulfoxide (DMSO) was added to dissolve formazan crystals within cells. The absorbance was then measured using Synergy™ Microplate Reader (BioTek Instruments, Winooski, VT) at a wavelength of 490 nm.

### 2.4. Colony Formation Assays

Colony formation was detected by crystal violet staining. Cells were seeded into 6-well plate at density of 5 × 10^4^ cells/well and incubated with 1–5 mg/mL TM for consecutive 7 days. Completed medium contained agents were changed every two days. Treated cells were fixed in 4% paraformaldehyde for 10 min and then stained with 0.1% crystal violet for 60 min. After three washes with phosphate buffered saline (PBS), cells were subsequently photographed. The experiments were repeated for three times.

### 2.5. Migration Assay

1 × 10^5^ cells/well were seeded into 6-well plates, cultured to over 90% confluence, and then scraped with a needle. After 24 h treatment with TM, migratory ability was evaluated by measuring the distance of cell migrating. The experiments were repeated for three times.

### 2.6. Apoptotic Rate Assay

Cells were seeded into 6-well plates at 4 × 10^5^ cells per well. After 24 h TM treatment at doses of 2.5 and 5 mg/mL, cells were stained with propidium iodide (PI) and Annexin V for 20 min at room temperature in darkness. The intensity of fluorescence was measured utilizing Muse™ Cell Analyzer from Millipore (Billerica, MA) following manufacturer's instructions. The experiments were repeated for three times.

### 2.7. Assessment of Caspases Activities

Cells incubated with 2.5 and 5 mg/mL of TM for 24 h were harvested using lysis buffer. After detection of protein concentrations, the activities of caspase-3, caspase-8, and caspase-9 were measured via related commercial kits (Nanjing Jiancheng Bioengineering Institute, Nanjing, China).

### 2.8. Assessment of Intracellular ROS Levels

2′-7′-Dichlorodihydrofluorescein diacetate (DCFH-DA, Sigma-Aldrich, USA) staining was applied to analyze intracellular ROS levels. After 6 h incubation with TM at doses of 2.5 and 5 mg/mL, cells were incubated with 10 *μ*M of DCFH-DA for 15 min. After three washes with PBS, the changes of green florescent intensity were examined under Nikon Eclipse TE 2000-S fluorescence microscope (20x; CCD camera; Nikon Instruments Inc., Japan).

### 2.9. Assessment of Mitochondrial Membrane Potential (MMP)

In our experiment, the changes of MMP were analyzed by 5,5′,6,6′-tetrachloro-1,1′,3,3′ tetraethylbenzimidazolylcarbocyanine iodide (JC-1; Calbiochem, USA) staining. Cells were seeded into 6-well plates at 1 × 10^5^ cells/well and incubated with 2.5 and 5 mg/kg of TM for 6 h, following with another 15 min exposure to 2 *μ*M of JC-1 at 37°C in darkness. After three washes, fluorescent color changes were analyzed using fluorescent microscopy under Nikon Eclipse TE 2000-S fluorescence microscope (20x; CCD camera; Nikon Instruments Inc., Japan).

### 2.10. HepG2- and SMMC-7721-Xenografted Tumor Mouse Models

The animal experimental protocol was approved by the Animal Ethics Committee of Jilin University. Six-week-old male BALB/c nude mice (Chares River Experimental Animal Technical Co., Ltd., Beijing, China) were housed in groups of three in clear plastic cages and maintained on a 12 h light/dark cycle at 23 ± 1°C with water and food available* ad libitum*.

HepG2 and SMMC-7721 cells were harvested at mid-log phase, and a total number of 5 × 10^6^ cells/100 *μ*L were subcutaneously injected into the right back near hind leg of each mouse. After 3 days, mice were divided into two groups (*n* = 3 each) randomly and treated with 1 g/kg of TM and 0.9% of saline solution every other day continuously for 14 days. Body weight and tumor dimensions were recorded before drug treatment. Tumor size was calculated as following equation: length (mm) × width (mm)^2^  × 0.5. At the end of the experiment, the liver and tumor tissues were collected and stored at −80°C.

### 2.11. Histopathological Examination

Liver tissues were immerged in 4% of paraformaldehyde for 24 h and dehydrated via 50%–100% ethanol step by step. After permeabilization with xylene, the samples were embedded in wax and cut into serial sections at 5 *μ*m thickness using microtome (Leica, Germany). Sections were immerged with fresh xylene for 10 min, hydrated with gradient ethanol from 100% to 70%, and finally washed with DD water [20]. Haematoxylin and eosin staining (H&E staining) was applied to examine histopathology of liver.

### 2.12. Western Blot Analysis

Collected cells, which were treated with 2.5 and 5 mg/mL of TM for 24 h, and collected tumor tissues were lysed by RIPA buffer (Sigma-Aldrich, USA) containing 1% protease inhibitor cocktail (Sigma-Aldrich, USA) and 2% phenylmethanesulfonyl fluoride (PMSF; Sigma-Aldrich, USA). Protein concentrations were determined using Bradford method. 40 *μ*g of proteins was separated by 10–12% SDS-PAGE gels and then transferred electrophoretically onto PVDF membranes. The transferred membranes were blocked in 5% bull serum albumin (BSA) for 4 h and then blotted with the following primary antibodies at 4°C overnight at dilution of 1 : 1000: cleaved poly(ADP-ribose) polymerase (cleaved-PARP) (ab32064), Bad (ab129192), Bax (ab7977), B-cell lymphoma-2 (Bcl-2) (ab32124), and glyceraldehyde-3-phosphate dehydrogenase (GAPDH) (ab8245) (Abcam, Cambridge, MA), followed by incubation with horseradish peroxidase-conjugated secondary antibodies diluted at 1 : 2000 (Santa Cruz, USA). ECL detection kits (Millipore, USA) were applied to detect chemiluminescence of blots, and the intensity was quantified by scanning densitometry using Image J (NIH, Bethesda, MD).

### 2.13. Statistical Analysis

Data are expressed as mean ± SD The statistics was analyzed by a one-way analysis of variance (ANOVA) followed with Dunn's test via SPSS 16.0 software (IBM corporation, Armonk, USA). The significance was chosen as *P* < 0.05.

## 3. Results

### 3.1. TM Caused Cell Damage in Hepatocellular Carcinoma Cells

**Figure 1:**
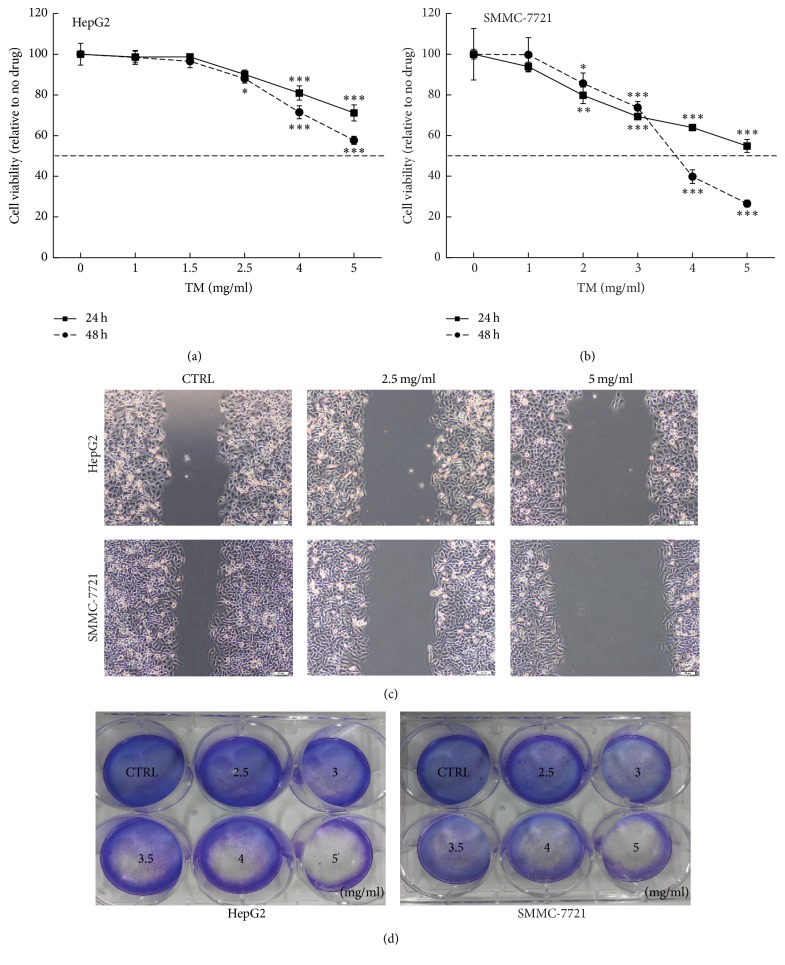
TM dose- and time-dependently reduced cell viability after 24 h and 48 h treatment in HepG2 (a) and SMMC-7721 (b) cells. Data are expressed as a percentage of that from corresponding control cells and means ± SD (*n* = 6). ^*∗*^ *P* < 0.05, ^*∗∗*^ *P* < 0.01, and ^*∗∗∗*^ *P* < 0.001 versus control cells. (c) TM inhibited the migration ability of hepatocellular carcinoma cells determined via a wound healing assay (10x; scale bar: 100 *μ*m) (*n* = 3). (d) TM suppressed hepatocellular carcinoma cell proliferation analyzed via crystal violet staining (*n* = 3).

A dose- and time-dependent cell viability reduction was noted in TM-treated cells, and the 48 h IC_50_ were 5.3 mg/mL and 3.4 mg/mL in HepG2 and SMMC-7721 cells, respectively (*P* < 0.05; Figures 1(a) and 1(b)). Inhibitory effects of TM on cell migration ability were analyzed by a wound healing assay. Compared with nontreated cells, the migration abilities in cells were strongly abolished by TM incubation at 2.5 and 5 mg/mL (Figure 1(c)). Moreover, TM abolished the clonogenic abilities of hepatocellular carcinoma cells which began at 3 mg/mL and showed completely blocked activities at 5 mg/mL (Figure 1(d)).

### 3.2. TM Induced Apoptosis in Hepatocellular Carcinoma Cells

**Figure 2:**
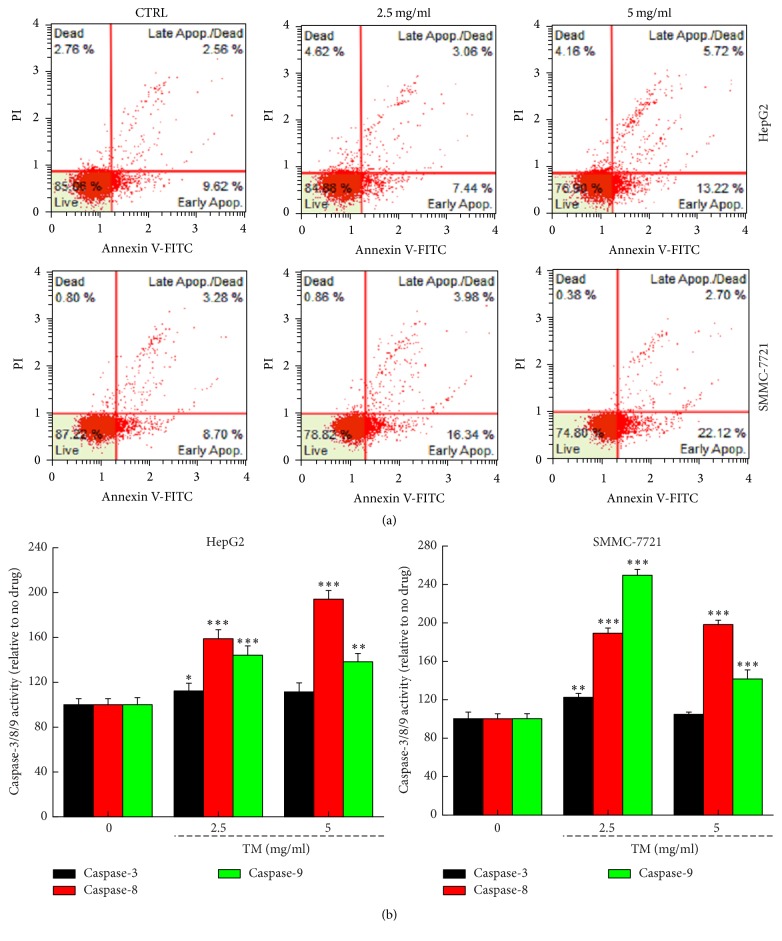
TM induced apoptosis in HepG2 and SMMC-7721 cells. (a) TM significantly enhanced the apoptotic rate in HepG2 and SMMC-7721 cells after 12 h incubation analyzing via flow cytometry (*n* = 3). (b) TM enhanced caspase-3, caspase-8, and caspase-9 activation in HepG2 and SMMC-7721 cells after 24 h exposure. Data are expressed as a percentage of that from corresponding control group and the means ± SD (*n* = 6). ^*∗*^ *P* < 0.05, ^*∗∗*^ *P* < 0.01, and ^*∗∗∗*^ *P* < 0.001 versus control cells.

12 h TM incubation enhanced the early and median apoptosis rate in HepG2 and SMMC-7721 cells at 2.5 and 5 mg/mL (Figure 2(a)). The maximum apoptosis rate reached 18.9% and 24.8% in HepG2 and SMMC-7721 cells, respectively (Figure 2(a)). The activation of caspase-3, caspase-8, or caspase-9 contributes to cell apoptosis and mitochondrial function [21, 22]. Compared with nontreated cells, TM increased 12.3%, 94.1%, and 44.2% activities of caspase-3, caspase-8, and caspase-9 in HepG2 cells, respectively (*P* < 0.05; Figure 2(b)). In SMMC-7721 cells, TM increased 22.4%, 89.1%, and 149.5% activities of caspase-3, caspase-8, and caspase-9, respectively (*P* < 0.01; Figure 2(b)).

### 3.3. TM Caused Apoptotic Alteration on Mitochondrial Function

**Figure 3:**
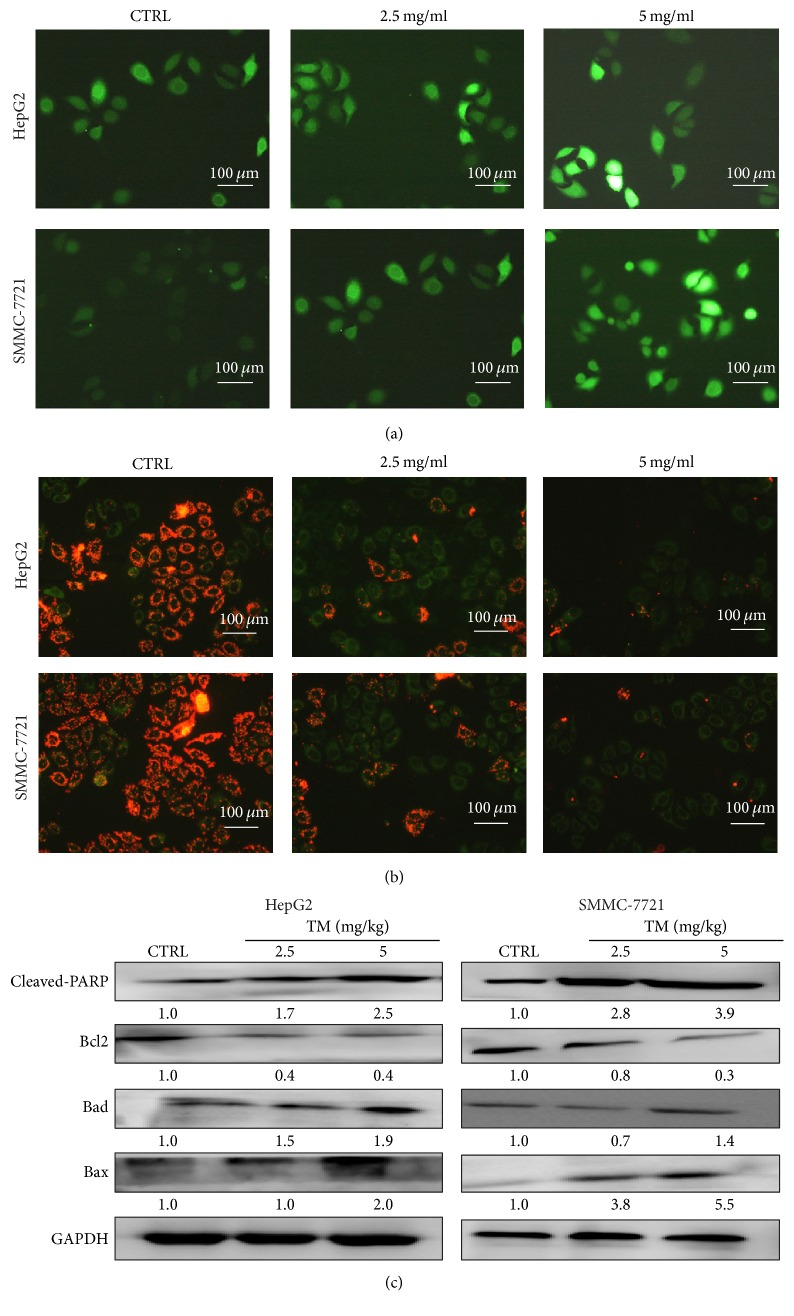
TM caused apoptotic alternations in mitochondrial function. (a) TM caused intracellular ROS accumulation in HepG2 and SMMC-7721 cells analyzing via DCFH-DA staining after 6 h incubation (20x; scale bar: 100 *μ*m) (*n* = 3). (b) TM induced MMP dissipation detecting by JC-1 staining after 6 h incubation (20x; scale bar: 100 *μ*m) (*n* = 3). (c) 24 h TM exposure enhanced the expressions of cleaved-PARP, Bax, and Bad and suppressed the levels of Bcl-2. Quantification data of protein expressions were normalized by corresponding GAPDH and the average fold of band intensity was marked, respectively (*n* = 3).

Intracellular ROS accumulation is considered as a typical event during cell apoptosis [23]. At 6 h exposure to 2.5 and 5 mg/mL of TM, intracellular ROS levels were strongly enhanced in HepG2 and SMMC-7721 cells indicated by the increment of green fluorescence intensity (Figure 3(a)).

Apoptotic alternation on mitochondrial was noted during cell apoptosis, which served as a target for tumor therapy [24]. In both HepG2 and SMMC-7721 cells, compared with nontreated cells, 6 h TM incubation at doses of 2.5 and 5 mg/mL strongly decreased MMP evidenced by the enhancement in green fluorescence (JC-1 monomers) and reduction in red fluorescence (JC-1 aggregates) (Figure 3(b)). Furthermore, 24 h TM incubation resulted in a strong increase on cleaved-PARP, Bad, and Bax levels, and a significant reduction on Bcl-2 expression in both HepG2 and SMMC-7721 cells (Figure 3(c)).

### 3.4. TM Inhibited HepG2- and SMMC-7721-Xenografted Tumor Growth

**Figure 4:**
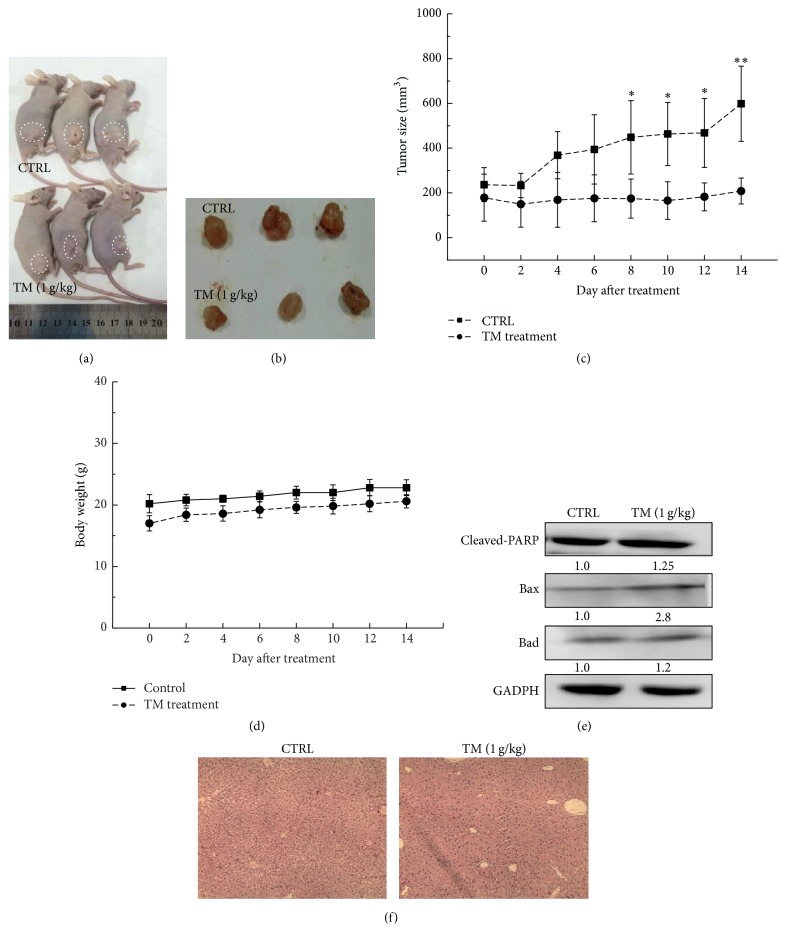
14-day TM (1 g/kg) administration suppressed tumor growth in HepG2-xenografted nude mouse models. (a) Tumor-possessing nude mice and (b) tumor tissues separated from CTRL and TM-treated groups. (c) Tumor volumes were measured every other day. Tumor size in the curve is expressed as mean ± SD (*n* = 3). ^*∗*^ *P* < 0.05 and ^*∗∗*^ *P* < 0.01 versus control group. (d) Mean (±SD) body weight of TM-treated and CTRL group (*n* = 3). (e) TM robustly enhanced the expressions of cleaved-PARP, Bad, and Bax in tumor tissues. Quantification data of protein expressions were normalized by corresponding GAPDH and the average fold of band intensity was marked, respectively (*n* = 3). (f) The histopathological examination of liver in tumor mouse models.

**Figure 5:**
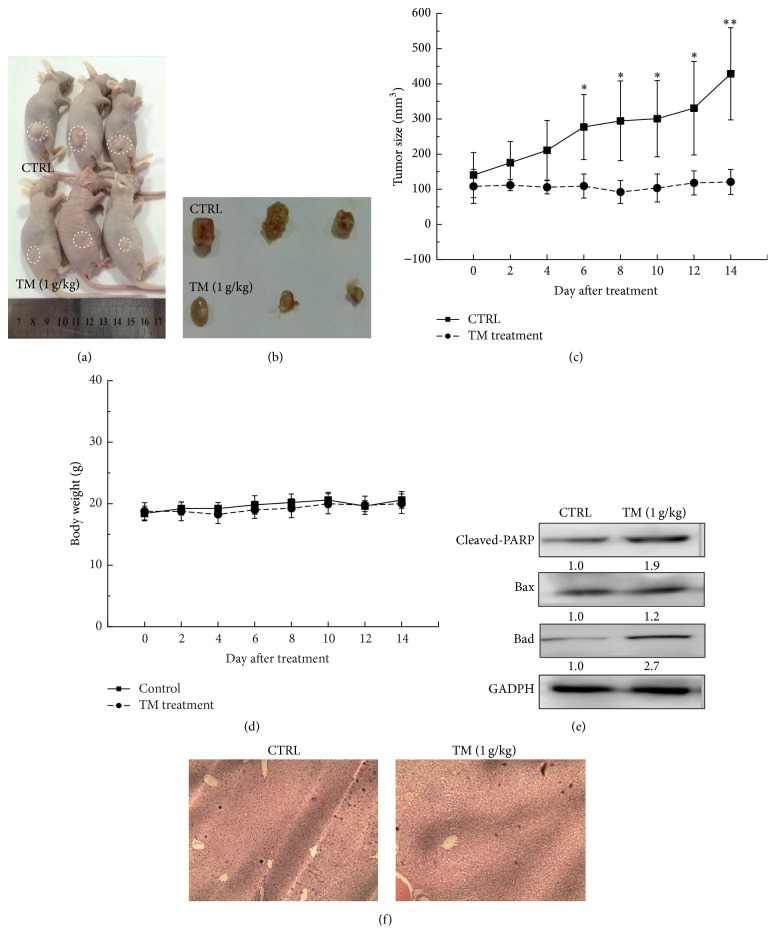
14-day TM (1 g/kg) administration suppressed tumor growth in SMMC-7721-xenografted nude mouse models. (a) Tumor-possessing nude mice and (b) tumor tissues separated from CTRL and TM-treated groups. (c) Tumor volumes were measured every other day. Tumor size in the curve is expressed as mean ± SD (*n* = 3). ^*∗*^ *P* < 0.05 and ^*∗∗*^ *P* < 0.01 versus control group. (d) Mean (±SD) body weight of TM-treated and CTRL group (*n* = 3). (e) TM robustly enhanced the expressions of cleaved-PARP, Bad, and Bax in tumor tissues. Quantification data of protein expressions were normalized by corresponding GAPDH and the average fold of band intensity was marked, respectively (*n* = 3). (f) The histopathological examination of liver in tumor mouse models.

HepG2 and SMMC-7721-xenografted tumor nude mouse models were applied to further confirm the antihepatocellular carcinoma effects of TM. 14-day TM (1 g/kg) administration robustly suppressed the HepG2 and SMMC-7721-xenografted tumor growth in nude mice without influence on their body weights (Figures 4(a), 4(b), and 4(d)) (Figures 5(a), 5(b), and 5(d)). Compared with CTRL group, at the 14th day, TM decreased 66.5% and 71.9% tumor size in HepG2 (*P* < 0.05; Figure 4(c)) and SMMC-7721-xenografted tumor mice (*P* < 0.05; Figure 5(c)), respectively. Similar to the results obtained in cell experiments, TM significantly enhanced the expressions of cleaved-PARP, Bax, and Bad in tumor tissues in both HepG2 (Figure 4(e)) and SMMC-7721-xenografted tumor mouse models (Figure 5(e)). Furthermore, H&E staining revealed that TM had no significant influence on liver function in HepG2- and SMMC-7721-xenografted tumor mice indicating TM is a safe agent (Figures 4(f) and 5(f)).

## 4. Discussion

We have studied the pharmacological activities of TM for years and confirmed that TM possesses antifatigue effects related to the modulation of AMPK linked antioxidation pathway [25]. Recently, our data show that TM strongly enhances immunity of cyclophosphamide-induced immunosuppressed mouse models associated with nuclear factor kappa B signaling. Based on these encouraging results, in the present experiments, the antihepatocellular carcinoma effects of TM were demonstrated in HepG2 and SMMC-7721 cells and related xenografted tumor mouse models. TM, a traditional Chinese herb medicine, contains multieffective components, which suggest multitargets in molecule signaling including apoptosis. Combining with previous data, the regulation of immune system may be involved in TM-mediated proapoptotic effects. Furthermore, this “systemic targeting” will help TM to show its pharmacological functions in a much natural way, so that fewer adverse effects are expected. Moreover, as a folk tonic food, TM has been practiced by Asian people for years, further emphasizing its safety. In our acute toxic test, the safety of TM was confirmed suggesting no significant influences on animal behaviors and body organs (data not shown). Moreover, the crude nature of TM may explain its non-dose-dependent manner observed in the present experiments, which is similar to other herbal medicines [26].

In our study, TM decreased cell viability, increased ROS levels, and caused mitochondrial apoptotic alterations. Mitochondrial dysfunction is related to cell apoptosis and is considered as a target for anticancer drugs [27]. In hepatocellular carcinoma cells, TM not only reduced the dissipation of MMP, but also suppressed Bcl-2 levels and enhanced the expressions of Bax and Bad. Bcl-2, an integral membrane protein located in the outer membrane of mitochondria, is known as a biomarker of mitochondria dysfunction [28]. Apoptosis relies on the balance between antiapoptotic (Bcl-2) and proapoptotic (Bax) proteins. Bax, a proapoptotic protein, induces the threshold at which mitochondria release Cyt C to promote cell death [29]. Similarly, in another experiment, we confirmed that* C. militaris* induces mitochondrial apoptosis in MCF-7 and HepG2 cells related to modulation on Bcl-2 family protein expressions [30]. Oxidative stress is another factor responsible for mitochondrial function [31], during which intracellular ROS accumulation is noted. In mitochondria-dependent apoptosis, a positive feedback loop between intracellular ROS and mitochondria is reported, and ROS is released from mitochondria into cytoplasm [32, 33]. Collectively, the antihepatocellular carcinoma effects of TM are related to mitochondrial apoptotic pathway; however, the relationship between mitochondrial function and oxidative stress needs further investigation.

On the other hand, the reduced MMP promotes the release of apoptotic factors from mitochondria to cytoplasm, and Cyt C is the most typical one, which leads to caspase-3 activation [34]. Caspase-3 is responsible for cell morphology and certain biochemical events including execution and completion of apoptosis [35]. As reported, caspase-3, activated by caspase-8 and caspase-9, cleaves vital cellular proteins, leading to apoptosis via mitochondria-dependent pathway [36]. In hepatocellular carcinoma cells, TM strongly enhanced the activation of caspase-3, caspase-8, and caspase-9. Caspase-8, located mostly at the mitochondria, may directly cause the loss of MMP when it undergoes dimerization and cleaves itself to be an activation form [37]. The activated caspase-8 can directly result in the cleavage of effector caspase in the cytosol [38]. All the results suggest that TM shows antihepatocellular carcinoma effects by inducing apoptosis via regulation caspase-family activities which may further target to mitochondria.

There are still some limitations in the present research. First, although we found the accumulation of intracellular ROS, its relationship with mitochondrial function is not clearly explained based on our data. Second, we only examined the antihepatocellular carcinoma effects of TM in HepG2 and SMMC-7721-xenografted tumor nude mouse models, which are hard to mimic the real satiation of tumor growth. Our ongoing experiments have focused on these limitations.

In conclusion, our study confirmed that TM induced apoptotic cell death in HepG2 and SMMC-7721 cells via caspase-dependent mitochondrial pathways. Our findings support further testing the antitumor effects of TM in other cell lines.
